# Supplementary material for: Explainable Risk Prediction of Post-Stroke Adverse Mental Outcomes Using Machine Learning Techniques in a Population of 1780 Patients
Source: Sensors (Basel). 2023 Sep 17;23(18):7946. doi: 10.3390/s23187946 (PMC10538068; doi:10.3390/s23187946)
Supplement: Supplementary file 1 [file sensors-23-07946-s001.zip › sensors-2585682-supplementary.pdf]

Supplementary Materials

Table S1. List of features collected and used for modelling

No.	Categories	Features	Remarks
1	Demographics	Gender	• Features are collected during admission date
2		Race	
3		Age at admission	
4		Marital Status	
5		Living Arrangement	
6		History of Depression	
7		Other Psy. History	
8		Family Psy. History	
9		Education Level	
10		Housing Type	
11		Occupation	
12		Alcohol Use	
13		Drug Use	
14		Smoking	
15	Stroke Information	Stroke Incidence	• Features are collected during admission date
16		Stroke Laterality	
17		Nature of Stroke	
18		Structure of Stroke	
19		Stroke Circulation	
20		Type of Stroke	
21	Medication History	Anti-depressants	• Searched for patient's lab results from 365 days before admission date to 3 days after admission date.
22		Anti-psychotics	
23		Benzodiazepines	
24		Dementia	
25		Mood Stabilizers	
26		Others	
27	Surgical History	AVM, aneurysm	• Searched for patient's lab results from 3 years before admission date to 3 days after admission date.
28		Craniectomy	
29		Craniotomy	
30		Cranioplasty	
31		Others	
32	Test scores	Abbreviated Mental Test (AMT)	• Features are collected during admission date
33		EuroQol 5-Dimensional – Visual Analog Scale (EQ5D-VAS)	
34		Functional Independence Measure (FIM) – Cognitive Score	
35		FIM – Motor Score	
36	Laboratory Results	Basophils	• Searched for patient's lab results from 365 days before admission date to 3 days after admission date.<br>• Selected the results closest to the admission date
37		Creatinine	
38		Eosinophils	
39		Haemoglobin	
40		Lymphocytes	
41		Sodium	
42		Potassium	
43		Red blood cell count	

44		Red blood cell width	
45		White blood cell count	
46	Psy. Intervention History	Psy. Intervention Services done	• Searched for patient's medication records from 3 years before admission date to 3 days after admission date.

Table S2. Descriptive Statistics of the study cohort

Features	Total (n = 1,780)		PSAMO (n = 285)		No PSAMO (n = 1,495)		p-value
	Results	Missing	Results	Missing	Results	Missing	
**Demographics**							
**Age at admission**	61 (53 – 70)	0	60 (52 – 68)	0	61 (54 – 70)	0	0.043*
**Gender, male (n, %)**	1,105, 62.08%	0	180, 63.16%	0	925, 61.87%	0	0.682
**Race, Chinese (n, %)**	1413, 79.94%	0	206, 72.28%	0	1217, 81.40%	0	0.002*
**Race, Malay (n, %)**	219, 12.3%		50, 17.54%		169, 11.3%		
**Race, Indian (n, %)**	137, 7.70%		29, 10.18%		109, 7.22%		
**Marital Status, Single (n, %)**	271, 15.22%	0	49, 17.19%	0	222, 14.85%	0	0.501
**Marital Status, Married (n, %)**	1186, 66.63%		189, 66.32%		997, 66.69%		
**Marital Status, Others (n, %)**	323, 18.15%		47, 16.49%		276, 18.46%		
**Living Arrangement, Alone (n, %)**	119, 6.69%	0	23, 8.07%	0	96, 6.42%	0	0.620
**Living Arrangement, With Family (n, %)**	1585, 89.04%		250, 87.72%		1335, 89.3%		
**Living Arrangement, Others (n, %)**	76, 4.27%		12, 4.21%		64, 4.27%		
**History of Depression (n, %)**	37, 2.08%	0	15, 5.26%	0	22, 1.47%	0	<0.001*
**Other Psy. History (n, %)**	25, 1.4%	0	8, 2.81%	0	17, 1.14%	0	0.028*
**Family Psy. History (n, %)**	23, 1.29%	0	5, 1.75%	0	18, 1.20%	0	0.451

**Education, Primary/No Formal (n, %)**	751, 42.19%	0	108, 37.89%	0	643, 43.01%	0	0.446
**Education, Secondary (n, %)**	685, 38.48%		119, 41.75%		566, 37.86%		
**Education, Diploma (n, %)**	218, 12.25%		36, 12.63%		182, 12.17%		
**Education, Degree or Higher (n, %)**	126, 7.08%		22, 7.72%		104, 6.96%		
**Housing Type, Rental/Dorms (n, %)**	260, 14.61%	80	41, 14.39%	22	219, 14.65%	58	0.038*
**Housing Type, HDB (non-rental) (n, %)**	1270, 71.35%		194, 68.07%		1076, 71.97%		
**Housing Type, Private (n, %)**	170, 9.55%		28, 9.82%		142, 9.5%		
**Household income, No Income (n, %)**	1228, 68.99%	0	243, 85.26%	0	985, 65.89%	0	<0.001*
**Household income (monthly), <$1000 (n, %)**	64, 3.6%		8, 2.81%		56, 3.75%		
**Household income (monthly), $1000 - $3000 (n, %)**	297, 16.69%		22, 7.72%		275, 18.43%		
**Household income (monthly), $3001 - $5000 (n, %)**	95, 5.34%		6, 2.11%		89, 5.95%		
**Household income (monthly), $5001 and above (n, %)**	96, 5.39%		6, 2.11%		90, 6.02%		
**Occupation, Not Working (n, %)**	803, 45.11%	34	127, 44.56%	1	676, 45.22%	33	0.181
**Occupation, Unskilled (n, %)**	371, 20.84%		59, 20.7%		312, 20.87%		
**Occupation, Skilled (n, %)**	572, 32.13%		98, 34.39%		474, 31.71%		
**Alcohol Use, Non-drinker (n, %)**	1497, 84.1%	93	231, 81.05%	22	1266, 84.68%	71	0.171
**Alcohol Use, Ex-drinker**	139, 7.81%		25, 8.77%		114, 7.63%		

**(n, %)**							
**Alcohol Use, Drinker (n, %)**	51, 2.87%		7, 2.46%		44, 2.94%		
**Drug Abuse, Yes (n, %)**	2, 0.14%	0	0, 0%	0	2, 0.16%	0	0.005*
**Smoking Use, Non-smoker (n, %)**	1179, 66.24%	174	178, 62.46%	32	1001, 66.96%	142	0.286
**Alcohol Use, Ex-smoker (n, %)**	329, 18.48%		62, 21.75%		267, 17.86%		
**Alcohol Use, Smoker (n, %)**	98, 5.51%		13, 4.56%		85, 5.69%		
**Stroke Information**							
**Stroke Incidence, First (n, %)**	894, 50.22%	0	140, 49.12%	0	754, 50.43%	0	0.011
**Stroke Incidence, First Clinical (n, %)**	572, 32.13%		72, 27.37%		494, 33.04%		
**Stroke Incidence, Recurrent (n, %)**	314, 17.64%		67, 23.51%		247, 16.52%		
**Stroke Laterality, Left (n, %)**	663, 37.25%	0	81, 28.42%	0	582, 38.93%	0	<0.001*
**Stroke Laterality, Right (n, %)**	1000, 56.18%		176, 61.75%		824, 55.12%		
**Stroke Laterality, Bilateral (n, %)**	117, 6.57%		28, 9.82%		89, 5.95%		
**Stroke Nature, Ischemic or Both (n, %)**	1290, 72.47%	0	192, 67.37%	0	1098, 73.44%	0	0.042*
**Stroke Nature, Haemorrhagic (n, %)**	490, 27.53%		93, 32.63%		397, 26.56%		
**Stroke Structure, Basal Ganglia (n, %)**	415, 23.31%	0	74, 25.96%	0	341, 22.81%	0	0.271
**Stroke Structure, Thalamus (n, %)**	162, 9.1%		30, 10.53%		132, 8.83%		
**Stroke Structure, Others (n, %)**	1203, 67.63%		181, 63.51%		1023, 68.43%		
**Stroke Circulation, Anterior (n, %)**	1095, 61.52%	0	184, 64.56%	0	911, 60.94%	0	0.504
**Stroke Circulation, Posterior (n, %)**	620, 34.83%		92, 32.28%		528, 35.32%		

**Stroke Circulation, Both (n, %)**	65, 3.65%		9, 3.16%		56, 3.75%		
**Stroke Type, Small Vessel Disease (n, %)**	1381, 77.58%	0	208, 72.98%	0	1173, 78.46%	0	0.099
**Stroke Type, Large Vessel Disease (n, %)**	241, 13.54%		49, 17.19%		192, 12.84%		
**Stroke Type, Others (n, %)**	158, 8.88%		28, 9.82%		130, 8.70%		
**Medication History (Last 1 year)**							
**Anti-depressants (n, %)**	261, 14.66%	0	57, 20%	0	204, 13.65%	0	0.005*
**Anti-psychotics (n, %)**	34, 1.91%	0	11, 3.86%	0	23, 1.54%	0	0.009*
**Benzodiazepines (n, %)**	51, 2.87%	0	14, 4.91%	0	37, 2.47%	0	0.024*
**Dementia meds (n, %)**	10, 0.56%	0	4, 1.4%	0	6, 0.4%	0	0.038*
**Mood Stabilizers (n, %)**	2, 0.11%	0	0, 0%	0	2, 0.13%	0	0.537
**Others (n, %)**	138, 7.75%	0	31, 10.88%	0	107, 7.16%	0	0.031*
**Surgical History (Last 3 Years)**							
**AVM, aneurysm (n, %)**	25, 1.40%	0	7, 2.46%	0	18, 1.2%	0	0.611
**Craniectomy (n, %)**	16, 0.9%	0	6, 2.11%	0	10, 0.67%	0	0.100
**Craniotomy (n, %)**	10, 0.65%	0	2, 0.7%	0	8, 0.54%	0	0.019*
**Cranioplasty (n, %)**	0, 0%	0	0, 0%	0	0, 0%	0	-
**Others (n, %)**	35, 1.97%	0	4, 1.4%	0	31, 2.07%	0	0.455
**FIM/EQ5D and other Tests**							
**AMT**	9 (8 – 10)	86	9 (8 – 10)	25	9 (8 – 10)	61	<0.001*
**EQ5D-VAS**	60 (50 – 80)	223	50 (40 – 70)	52	60 (50 – 80)	171	<0.001*
**FIM – Cognitive Score**	28 (21 – 33)	1	23 (16 – 30)	1	28 (21 – 33)	0	<0.001*
**FIM – Motor Score**	46 (32 – 57)	1	33 (23 – 49)	0	47 (35 – 58)	1	<0.001*
**Laboratory Parameters**							
**Basophils**	0.04 (0.02 – 0.05)	319	0.04 (0.03 – 0.06)	36	0.04 (0.02 – 0.05)	283	<0.001*
**Haemoglobin**	13.4 (12.3 – 14.5)	319	13.4 (12.2 – 14.5)	36	13.3 (12.3 – 14.4)	283	0.715
**Creatinine**	75 (61 – 93)	303	78 (62 – 97)	32	75 (60 – 93)	271	0.116
**Eosinophils**	0.16	319	0.17	36	0.16	283	0.168

	(0.08 – 0.27)		(0.09 – 0.28)		(0.08 – 0.27)		
**Potassium**	3.8<br>(3.6 – 4.1)	304	3.7<br>(3.5 – 4.1)	32	3.8<br>(3.6 – 4.1)	272	0.006*
**Lymphocytes**	1.67<br>(1.27 – 2.19)	319	1.73<br>(1.33 – 2.26)	36	1.66<br>(1.26 – 2.17)	283	0.287
**Red Blood Count Mean (SD)**	4.5<br>(0.65)	319	4.54<br>(0.66)	36	4.49<br>(0.65)	283	0.456
**Red Cell Distribution Width**	13.8<br>(13.2 - 14.6)	319	13.9<br>(13.4 – 14.7)	36	13.7<br>(13.2 - 14.6)	283	0.021*
**Sodium**	138<br>(136 – 140)	304	138<br>(136 – 140)	32	138<br>(136 – 140)	272	0.090
**White Blood Cells**	8.4<br>(6.8 – 10.2)	319	8.7<br>(7 – 11.3)	36	8.3<br>(6.8 – 10.13)	283	0.009*
**Psy. Intervention History (Last 3 years)**							
**Psy Intervention Services (n, %)**	107,<br>6.01%	0	19,<br>6.67%	0	88,<br>5.89%	0	0.611

All figures are in median (IQR) unless specified
**Statistically significant at α = 0.05*
